# Monosaccharide-Responsive Phenylboronate-Polyol Cell Scaffolds for Cell Sheet and Tissue Engineering Applications

**DOI:** 10.1371/journal.pone.0077861

**Published:** 2013-10-22

**Authors:** Rachamalla Maheedhar Reddy, Akshay Srivastava, Ashok Kumar

**Affiliations:** Department of Biological Sciences and Bioengineering, Indian Institute of Technology Kanpur, Kanpur, India; RMIT University, Australia

## Abstract

Analyte-responsive smart polymeric materials are of great interest and have been actively investigated in the field of regenerative medicine. Phenylboronate containing copolymers form gels with polyols under alkaline conditions. Monosaccharides, by virtue of their higher affinity towards boronate, can displace polyols and solubilize such gels. In the present study, we investigate the possibility of utilizing phenylboronate-polyol interactions at physiological pH in order to develop monosaccharide-responsive degradable scaffold materials for systems dealing with cells and tissues. Amine assisted phenylboronate-polyol interactions were employed to develop novel hydrogel and cryogel scaffolds at neutral pH. The scaffolds displayed monosaccharide inducible gel-sol phase transformability. *In vitro* cell culture studies demonstrated the ability of scaffolds to support cell adhesion, viability and proliferation. Fructose induced gel degradation is used to recover cells cultured on the hydrogels. The cryogels displayed open macroporous structure and superior mechanical properties. These novel phase transformable phenylboronate-polyol based scaffolds displayed a great potential for various cell sheet and tissue engineering applications. Their monosaccharide responsiveness at physiological pH is very useful and can be utilized in the fields of cell immobilization, spheroid culture, saccharide recognition and analyte-responsive drug delivery.

## Introduction

Tissue engineering and cell sheet engineering deal with the culture and recovery of biological cells in the form of tissues and cell sheets, respectively, intended for various applications of regenerative medicine including *in vivo* tissue reconstruction [1]–[3]. Tissue engineering applications often involve a three-dimensional (3-D) porous scaffold that allows mammalian cell adhesion, proliferation and regeneration into tissues of desired sizes and predefined shapes. An ideal scaffold should possess interconnected macroporous structure to promote unhindered cell infiltration and proliferation into tissues [4]. Such a scaffold structure also enables free exchange of nutrients and oxygen between cells and their environment [5]. The scaffold should also possess adequate mechanical strength in order to accommodate growing tissue without any mechanical failure [4]. Cryogels are open supermacroporous structures constructed through the cryogenic polymerization and/or crosslinking of the gel-forming precursors in their moderately frozen solutions at sub-zero temperatures [6]–[8]. The interconnected macroporous structure, high surface area and superior mechanical strength of cryogels present them as ideal tissue engineering scaffolds [8], [9]. Cell sheet engineering, a second generation cell based therapy, views a tissue as an intimate histological association of cell sheets either of same or different cell types into a 3-D lattice. It aims to achieve tissue reconstruction through assembly of individual cell sheets [2], [10]–[13]. Such applications typically prefer special two-dimensional (2-D) scaffolds or surfaces over 3-D structures.

Biodegradability is a key issue for tissue engineering scaffolds and implants where the degradation of scaffold allows voids for tissue in-growth [14]. The degradation rate of scaffold should match the production of extra cellular matrix by cells, promoting tissue growth in 3-D as well as resulting in mechanical stability. Synthetic polymers constitute a major portion of existing cell culture scaffolds. The majority of these polymers do not possess biodegradability on their own. This seriously limits their widespread usage as scaffold materials. Therefore, a synthetic polymeric material with analyte responsive degradability has great potential. Poly(*N*-isopropylacrylamide) (PNIPAAm), a well known thermoresponsive polymer, exhibits abrupt reversible hydrophilic to hydrophobic macromolecular transition at its lower critical solution temperature (LCST) which is around 32°C. The confluent cell monolayer grown on culture dish grafted with PNIPAAm chains gets detached from the surface on lowering the culture temperature below 32°C [11]–[13]. This approach remains one of the very few viable cell sheet engineering approaches in practice. However, even this approach demands highly skilled techniques and confined to very few research groups. A polymeric 2-D scaffold that degrades in response to an analyte and thereby allows the detachment of confluent cell monolayer from its surface can serve as a potential alternative to the existing cell sheet engineering scaffolds.

**Figure 1:**
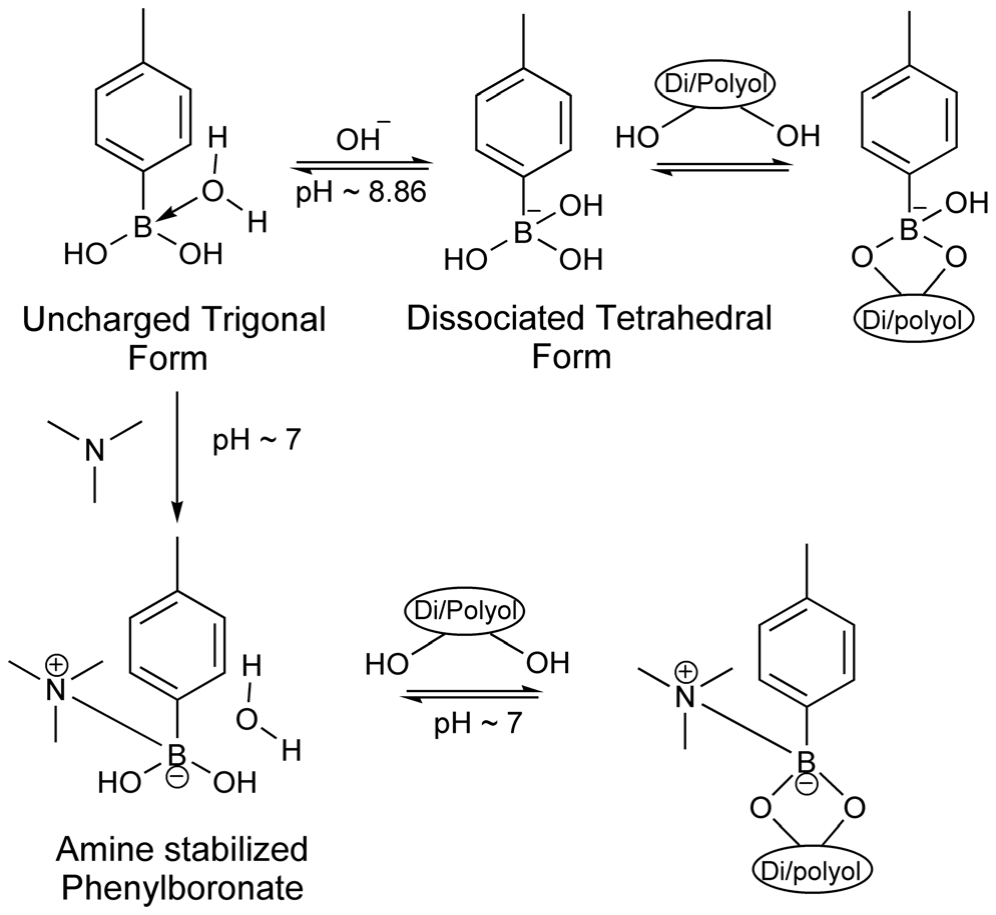
Phenylboronate-cis diol chemistry. In aqueous solutions, phenylboronate (PBA) exists in equilibrium between uncharged trigonal and charged tetrahedral forms. Anionic PBA forms reversible covalent interactions with 1,2- or 1,3-cis diols at alkaline pH (>8.8). A tertiary amine protects boronate moiety from the nucleophilic attack by water molecule and promotes PBA-cis diol interactions at neutral pH.

Phenylboronic acid (PBA) forms specific and reversible covalent interactions with 1,2- or 1,3- cis diol containing compounds. In the aqueous medium, PBA exists in equilibrium between the uncharged trigonal form and the anionic tetrahedral form with a pK_a_ around 8.8 [15]. The uncharged PBA-diol interactions are unstable because of their high susceptibility to hydrolysis. On the other hand, anionic PBA forms stable interactions with diols [15]–[17] (Fig. 1). Phenylboronate containing copolymers (PBCCs) form complex gels with poly(vinyl alcohol) (PVA) via intermolecular multipoint monodiols only under alkaline conditions and the fraction of anionic PBA available in the copolymer at physiological pH is not sufficient enough to form gels [18]–[21]. Such complex gels have been investigated as glucose sensitive drug delivery systems [19], [20], [22] and fructose sensitive mucosal lumen occlusive gels [23], since the monosaccharides with their higher affinity to boronates competitively bind and disintegrate the gels. However, gels formed under alkaline conditions are unable to deal with cells and tissues. Few studies reported that the amino groups introduced in to the copolymer stabilize the boronate-diol complex at neutral pH by protecting it from the nucleophilic attack by the water molecule [20], [22], [24] (Fig. 1).

The present study aims at using amine assisted PBCC-PVA interactions to impart biodegradability like characteristics on to the non-degradable synthetic polymers and to develop gel-sol phase transformable scaffolds for tissue engineering and cell sheet engineering applications. In this study, we employed *N,N*-dimethylaminoethylmethacrylate (DMAEMA) as flanking tertiary amine provider, either 4-vinylphenylboronic acid (VPBA) or *N*-acryloyl-3-aminophenylboronic acid (AAPBA) as phenylboronate ligand and either *N*-isopropylacrylamide (NIPAAm) or acrylamide (AAm) or *N,N*-dimethylacrylamide (DMAAm) as the monomer that forms majority of the polymer back bone to synthesize three different PBCCs (terpolymers). We were able to synthesize PBCC-PVA hydrogel films at physiological pH and culture cells on such films. The monosaccharide inducible gel-sol phase transformability of such gels is then exploited to recover the cultured cells. We further synthesized open macroporous PBCC-PVA cryogels and studied their monosaccharide inducible degradability as well as their ability to support cell adhesion, viability and growth.

## Materials and Methods

### Materials

All the chemicals used were of reagent grade. *N*-isopropylacrylamide (NIPAAm) was purchased from Acros Organics (New Jersey, USA). Acrylamide (AAm) and dimethylsulfoxide (DMSO) were bought from Merck (Mumbai, India). *N,N*-dimethylacrylamide and acryloyl chloride were products of Fluka (Buchs, Switzerland). *N,N*-Dimethylaminoethylmethacrylate (DMAEMA) was a product of Alfa Aesar (Heysham, UK). 4-Vinylphenylboronic acid (VPBA) was bought from Aldrich (St. Louis, USA). Ammonium persulfate (APS) and *N,N,N',N'*-tetramethylethylenediamine (TEMED) were purchased from Sisco Research Laboratories Pvt. Ltd. (Mumbai, India). Dulbecco's Modified Eagle's Medium (DMEM), penicillin - streptomycin solution, trypsin, 3-(4,5-dimethylthiazol-2-yl)-2,5-diphenyltetrazolium bromide (MTT), fluorescein diacetate (FDA), 4',6-diamidino-2-phenylindole (DAPI) and trypan blue were purchased from Sigma (St. Louis, USA). 3-Aminophenylboronic acid was bought from Varata Chemicals Ltd. (Mumbai, India). Fetal bovine serum (FBS) was a product of Gibco (Grand Island, USA). Polyvinyl alcohol (PVA), fructose and dextrose were purchased from s. d. fine-chem limited (Mumbai, India).

### Synthesis of poly(NIPAAm-*co*-VPBA-*co*-DMAEMA) (NVDT)

NIPAAm (900 mg), 50 mg of VPBA and 53.5 µl of DMAEMA were dissolved in 10 ml of degassed 100 mM NaOH solution (pH∼13) i.e., a total monomer concentration of 10% ((w/v), and further on throughout the experimental section for the solute concentration). The solution was bubbled with nitrogen for 20 min and free radical polymerization was initiated by adding 15 mg of APS and 19.5 µl of TEMED. The reaction vial was immediately filled with nitrogen and tightly sealed. The polymerization was terminated after 18 h by thermo-precipitating the terpolymer from the solution by heating at 60°C for 5 min. The polymer was re-dissolved in 5 ml of double distilled (DD) water by magnet aided stirring at 5°C followed by thermoprecipitation to remove unreacted monomers. The washing was repeated for one more time and the precipitated terpolymer was freeze dried. Further, a terpolymer solution (2.5%) was prepared with pH adjusted to neutrality by using 1 M Hydrochloric acid, autoclaved at 15 lb pressure for 20 min and cooled to 4°C. The copolymerization was confirmed through Fourier Transform Infrared Spectroscopy (FT-IR) (Bruker Vertex 70 FT-IR Spectrometer).

### Synthesis of poly(AAm-*co*-VPBA-*co*-DMAEMA) (AVDT) and poly(DMAAM-*co*-VPBA-*co*-DMAEMA) (DVDT)

AAm (800 mg), VPBA (100 mg) and DMAEMA (107 µl) were dissolved in 10 ml of degassed 100 mM NaOH solution (pH ∼13, total monomer ∼10%) followed by nitrogen bubbling for 20 min. Polymerization was initiated with the addition of APS (15 mg) and TEMED (19.5 µl). After 18 h of polymerization the solution was dialyzed against 100 fold excess deionized water using a 15,000 MW cutoff membrane for 48 h with water being replaced once after 24 h. After dialysis the final volume of the solution was made to 40 ml with double distilled water and pH was adjusted to ∼7.4. The solution was then autoclaved at 15 lb pressure for 20 min and cooled to room temperature. DVDT was synthesized essentially in the same way as described for AVDT except that DMAAm was used in the place of AAm. At room temperature, DVDT exhibits a gel like morphology due to physical crosslinking [28] and it was converted into the sol state by heating to 65°C for 15 min just before its use.

### Preparation of gel-sol phase transformable hydrogels

Three different hydrogels i.e., NVDT-PVA, AVDT-PVA and DVDT-PVA, were synthesized by mixing equal volumes (1 ml) of the respective terpolymer (2.5%) and PVA (2.5%) solutions in glass vials. Gel to sol phase transition of the hydrogels was confirmed by the addition of 1 ml of either fructose or glucose solutions with concentrations ranging from 100 mM to 1 M.

### Cell culture and recovery using 2-D hydrogel films

Sterilized PVA solution (2.5%, 100 µl) and NVDT/AVDT/DVDT solution (2.5%, 100 µl) were added in to each well of a 24 well non treated tissue culture plate and mixed thoroughly with sterile microtip until a visible hydrogel film is developed. The wells are then undisturbed for 6 h to allow the gels to settle. NIH3T3 fibroblasts (4×10^4^ cells/ml) in 1 ml of DMEM were seeded on to each hydrogel containing well and incubated at 4°C to allow the sedimentation of cells on to the gels. After 30 min at 4°C, the wells were incubated at 37°C in an incubator with humidified 5% CO_2_ atmosphere. The DMEM used, consisted of 25 mM glucose and supplemented with 10% FBS and 1% penicillin- streptomycin solution. Medium was changed every 2–3 days. After 15 days in culture, DMEM was replaced with sterile 200 mM fructose solution (1.5 ml) and the culture plate was swung at 60 rpm in an incubator shaker for 40–45 min. The wells were observed under inverted microscope at regular intervals during this period.

### Synthesis of phase transformable cryogels

NIPAAm (900 mg), VPBA (100 mg) and TEMED (39 µl) were completely dissolved in 10 ml of degassed 100 mM NaOH solution (pH∼13, total monomer ∼10%) and nitrogen bubbled for 20 min. PVA (500 mg) was dissolved in 9.5 ml of degassed 100 mM NaOH by heating at 90°C under magnetic stirring for 30–45 min and cooled to room temperature. APS (30 mg) dissolved in 0.5 ml of degassed water was added to PVA solution. Both the monomer-TEMED and PVA-APS solutions, pre-cooled to 4°C, were well mixed and immediately transferred in to 2.5 ml disposable syringes kept in the cryostat (Lauda, Proline PR 1840, Germany) maintained at –20°C. The final 20 ml solution consisted of 5% monomer and 2.5% PVA. After 18 h of cryogelation, NVP cryogels were removed from the cryostat and freeze dried. AVP cryogels were prepared in essentially the same manner as described above except that 900 mg of AAm was used instead of NIPAAm.

*N*-Acryloyl-3-aminophenylboronic Acid (AAPBA) was synthesized as described elsewhere [21]. AAPBA (100 mg) was dissolved in 10 ml of double distilled (DD) degassed water (pH∼7.4) at 60 °C for 15–20 min followed by cooling to room temperature. AAm (800 mg), DMAEMA (107 µl) and TEMED (19.5 µl) were completely dissolved in the AAPBA solution (a total monomer concentration of 10%) and nitrogen bubbled for 20 min. PVA (500 mg) was dissolved in 9.5 ml of DD degassed water (pH∼7.4) by heating at 90°C under magnetic stirring for 30–45 min, cooled to room temperature and then 15 mg of APS dissolved in 0.5 ml of degassed water was added to it. Both the monomer-TEMED and PVA-APS solutions were pre-cooled, mixed together and immediately transferred in to 2.5 ml disposable syringes kept in the cryostat maintained at –20°C. After 18 h of cryogelation, AADP cryogels were freeze dried.

Pore structure and surface morphology of the cryogels were studied with the help of scanning electron microscope (SEM, FEI Quanta 200). Freeze dried cryogel sections were coated with gold using a sputter coating unit (Vacuum Tech, Bangalore, India). The SEM was operated under high vacuum, at 20 KV with spot size setting of 4.5. The pore diameter was measured using image analysis software associated with the microscope.

### Compression testing analysis of cryogels

Compression tests were conducted on cylindrical samples of NVP cryogel (of height 11 mm and cross section 50.265 sq.mm), AVP cryogel (of height 10.3 mm and cross section 51.53 sq.mm) and AADP cryogel (of height 17 mm and cross section 56.745 sq.mm). The cylindrical samples were subjected to uniaxial stress using Instron 1195 computerized testing machine with 100 KN load cell under displacement control at the rate of 1 mm/min. Given a cryogel sample of length *l* and cross sectional area *A* subjected to a uniaxial compressive load of *F*, the modulus of elasticity (*λ*) was determined from the elastic deformation portion of stress-strain graph using the following formula:
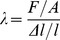



### Fructose induced disintegration of cryogels

For sugar induced disintegration studies of cryogels, three different fructose concentrations i.e., 100 mM, 500 mM and 1 M, were employed. Phosphate buffer (10 mM) without any sugar was used as control. Cryogel samples with predetermined dry weights (∼110 mg–147 mg) were transferred into 50 ml Tarsons centrifuge tubes each containing 10 ml of fructose- phosphate buffer solutions. The tubes were incubated at 37°C with a gentle shaking of 60–90 rpm. The fructose-phosphate buffer solution was changed into fresh one for every 5 days. All the studies were carried out in duplicate. After 15 days, the cryogel samples were taken out and freeze dried for 48 h and weighed. The percentage reduction in dry weight of each cryogel sample in response to the specific fructose concentration was plotted against time.

### Cell culture using 3-D cryogel scaffolds

Cylindrical cryogel sections of diameter, 8 mm and thickness, 1 mm were washed with excess amounts of deionized water to remove the traces of alkalinity (in the case of VPBA containing cryogels) and unreacted monomers. The cryogel sections are sterilized by placing each section in 2 ml of 2% penicillin - streptomycin solution for 48 h. Then the sections are transferred in to a 24-well tissue culture plate, one section in each well. NIH3T3 fibroblasts (5.5×10^4^ cells) suspended in 100 µl of DMEM were directly seeded on to the scaffolds followed by the addition of 1 ml of DMEM supplemented with 10% FBS and 1% penicillin-streptomycin solution. The cell seeded scaffolds were incubated at 37°C in an incubator with humidified 5% CO_2_ environment. The medium was replaced with fresh one for every 2 days. After 12 days of cell culture, the medium was removed and the scaffolds are washed with 1.5 ml of cold PBS. Then the cell scaffolds were stained with 500 µl of FDA (5 µg/ml, working concentration), a vital dye, for 5 min and 200 µl of DAPI (0.1 µg/ml, working concentration) for 15 min. After 15 min, the dyes were pipetted out and the scaffolds were gently washed twice with 1.5 ml of cold PBS in order to remove excess dye. The cell distribution and viability in FDA/DAPI stained cryogel sections were immediately observed under fluorescence microscope (Zeiss, Axioscope 2 plus, Germany). The images of DAPI stained cells (both viable and non-viable) were taken under DAPI filter, those of FDA stained viable cells were taken under FITC (Fluorescein isothiocyanate) filter. At the same magnification and specimen position, the images of scaffold were taken under bright field. All the three individual images were then merged into a RGB image using Adobe Photoshop.

Thin cryogel sections (of 8 mm diameter and 3 mm thickness) sterilized with 2% antibiotic solution for 48 h were directly seeded with NIH3T3 fibroblasts (1.5×10^5^) suspended in 200 µl of DMEM. DMEM (1 ml) supplemented with 10% FBS and 1% penicillin-streptomycin solution was added in to each scaffold containing well. NIH3T3 fibroblasts (1.5×10^5^ cells) seeded on to 12-well tissue culture polystyrene (TCPS) plates were used as control. Both the cell loaded cryogels and controls were incubated at 37°C in an incubator with humidified 5% CO_2_ atmosphere. At each 2-day interval, the culture medium was removed from the test wells and gently washed with cold PBS (100 mM). The serum free DMEM medium (0.5 ml) containing MTT at a working concentration of 0.5 mg/ml, was added to the wells and incubated at 37°C for 5 h. The MTT reagent in each well was gently replaced with 1.5 ml of dimethylsulfoxide (DMSO), which dissolves intracellular purple formazan crystals to give blue-violet colored solution which was read by a spectrophotometer at 570 nm. The absorbance at 570 nm was plotted against time to assess cell activity and proliferation within cryogel sections.

## Results

### Synthesis of terpolymers

Three different terpolymers poly(NIPAAm-*co*-VPBA-*co*-DMAEMA) (NVDT), poly(AAm-*co*-VPBA-*co*-DMAEMA) (AVDT) and poly(DMAAm-*co*-VPBA-*co*-DMAEMA) (DVDT) were synthesized through free radical polymerization in alkaline aqueous medium. NVDT exhibited thermo- and pH- dual responsiveness. In contrast to the sharp LCST of NIPAAm, NVDT exhibited a gradual thermo-responsiveness over the range of 29°C – 34°C (pH∼7.4) (Fig. S1). At 27°C, the terpolymer was insoluble in water at pH < 4–5. The copolymerization was confirmed through FT-IR spectroscopy (Fig. S2).

### Preparation of gel-sol phase transformable hydrogels

**Figure 2:**
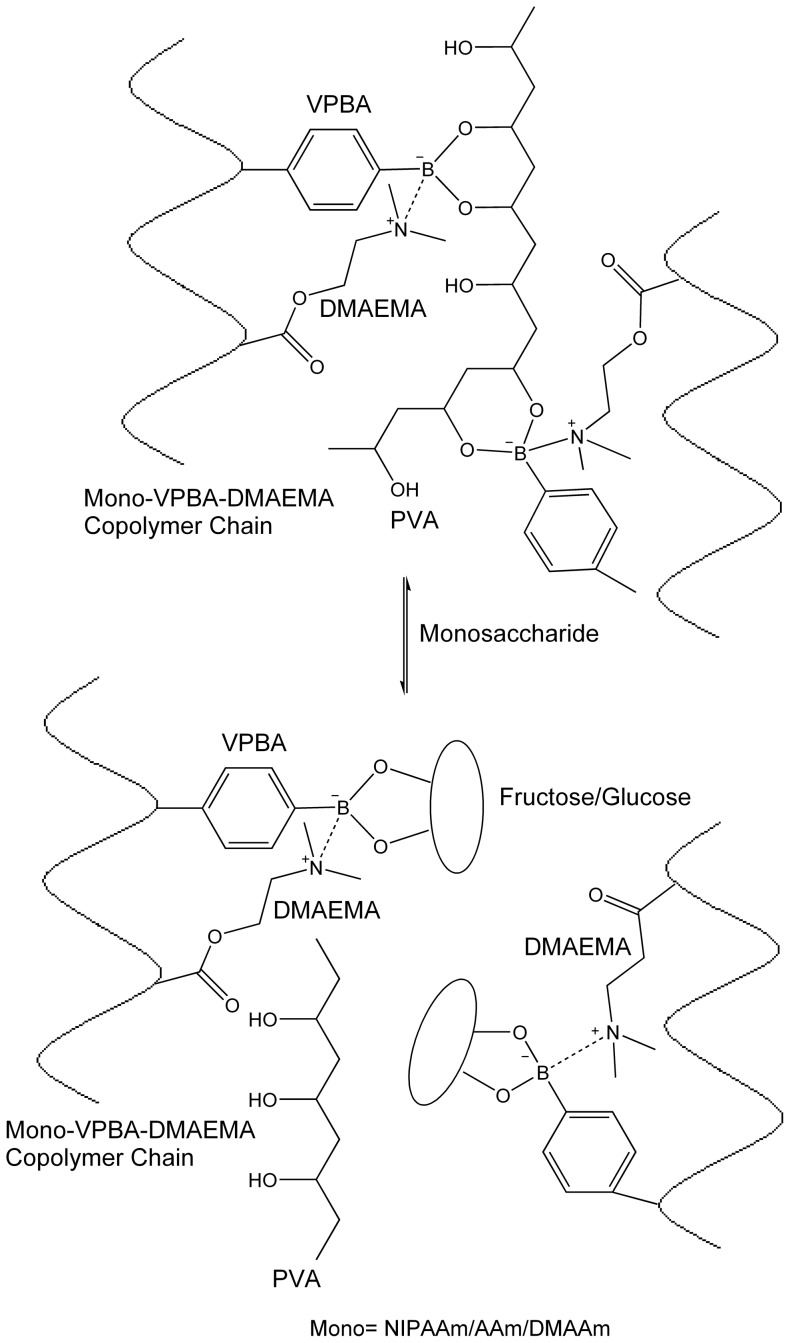
Terpolymer-poly(vinylalcohol) (PVA) complexation and fructose induced gel-sol phase transition at physiological pH. Phenylboronate containing copolymer (PBCC) forms complex gels with polyols like PVA *via* intermolecular multipoint monodiols under alkaline conditions. A tertiary amine introduced into PBCC structure promotes multipoint monodiols at neutral pH. Monosaccharides such as fructose, by virtue of their higher affinity towards phenylboronate, competitively displace PVA from monodiol interactions and disintegrate the gels.

**Figure 3:**
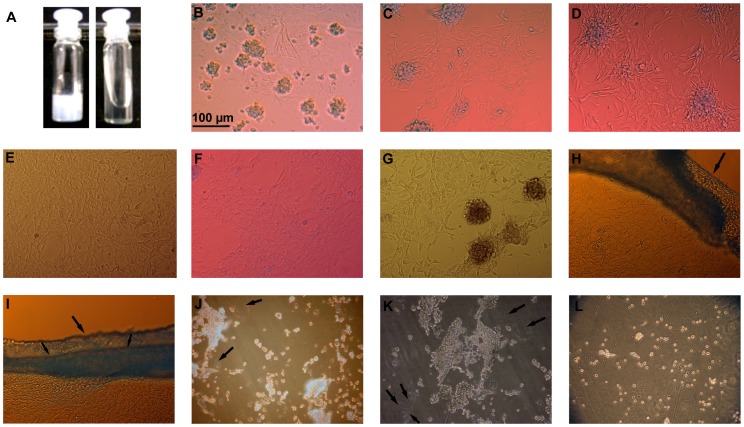
Culture and Recovery of NIH3T3 fibroblasts on terpolymer-poly(vinylalcohol) (PVA) hydrogel films. Fig. 3A shows fructose induced gel-sol transition of AVDT-PVA hydrogel. Figs. 3B-E shows culture of NIH3T3 fibroblasts on DVDT-PVA hydrogel films at day 2, 6, 8 and 12, respectively. Initial aggregation of fibroblasts at specific positions on the gel can be seen in Fig. 3B. After day 2, the fibroblasts have begun to spread out on to the surrounding gel (Figs. 3C, D). On DVDT-PVA hydrogel, the growth has reached to confluence by day 12 (Fig. 3E). Figs. 3F, G show the fibroblast growth after 12 days in culture on AVDT-PVA and NVDT-PVA hydrogels, respectively. Figs. 3H, I show detached fibroblast monolayer from AVDT-PVA gel at the edges (arrows) on fructose (200 mM) treatment with stirring (60 rpm) for 20 min. Continued fructose treatment has resulted in the recovery of individual and small clumps of cells. The gel remnants are indicated by arrows (Figs 3J-L).

The terpolymer solutions (2.5% w/v) formed insoluble gels with PVA solution (2.5% w/v) at neutral pH. The tertiary amine of DMAEMA incorporated into the copolymer interacts with boronate of VPBA and stabilizes boronate-diol complex by protecting it from surrounding water molecules at physiological pH. Owing to its polymeric nature PVA forms intermolecular multipoint monodiols between different terpolymer chains and crosslink them in solution resulting in a hydrogel (Fig. 2). The gels were then solubilized through administration of fructose and glucose solutions. Monosaccharides, because of their extremely high affinity towards phenylboronate as compared to PVA (association constants, K_ass_, of fructose and PVA towards borate are ∼1700 M^−1^ [25] and ∼2.8 M^−1^ [26], respectively), competitively displace PVA (Fig. 2). As a result, hydrogel gets disintegrated into sol state (Fig. 3A). The dissolution rate was observed to depend on the type and concentration of monosaccharide employed. The gel to sol transition was very rapid and of the order of few seconds where 500 mM-1 M monosaccharide solutions were employed while the transition was relatively slow (∼45–50 min) with 100 mM–200 mM solutions.

### Cell culture and recovery using 2-D hydrogel films

Thin films of all the three types of terpolymer-PVA hydrogels were prepared by mixing equal amounts of the respective terpolymer and PVA solutions (100 µl each) into each well of a 24-well tissue culture plate. NIH3T3 fibroblast cells (4×10^4 ^cells/ml) were seeded and allowed to sediment on to the hydrogels in Dulbecco's Modified Eagle's Medium (DMEM). The plates were then incubated at 37°C in humidified 5% CO_2_ environment. Following cell seeding, within the first 24 h, the cells migrated and formed aggregates at specific positions on the hydrogel (Fig. 3B). Within these aggregates the cells remained viable and round in morphology. After 6-8 days in culture, the cells located at the bottom of the aggregates started spreading out onto the surrounding hydrogel (Figs. 3C, D). The cell spreading slowly progressed from bottom to the top of the aggregates with time. DVDT-PVA (Fig. 3E) and AVDT-PVA (Fig. 3F) hydrogels attained confluence by day 12 whereas NVDT-PVA hydrogels exhibited relatively slow cell spreading and proliferation and became confluent by day 15 (Fig. 3G). After 15 days in culture, we attempted to recover the cells by dissolving AVD terpolymer-PVA hydrogels. The medium in the wells was replaced with 1.5 ml of sterilized solution containing 0.2 M fructose and 0.15 M sodium chloride (NaCl) and swung gently at 60 rpm. After 20 min of stirring, gel got solubilized and the fibroblast monolayer was detached from the gel from the corners of the well (Fig. 3H, J). Continued stirring for 15 more minutes resulted in the breakdown of sheet and recovery of small cell aggregates and individual cells (Fig. 3J-L).

### Synthesis of phase transformable cryogels

**Figure 4:**
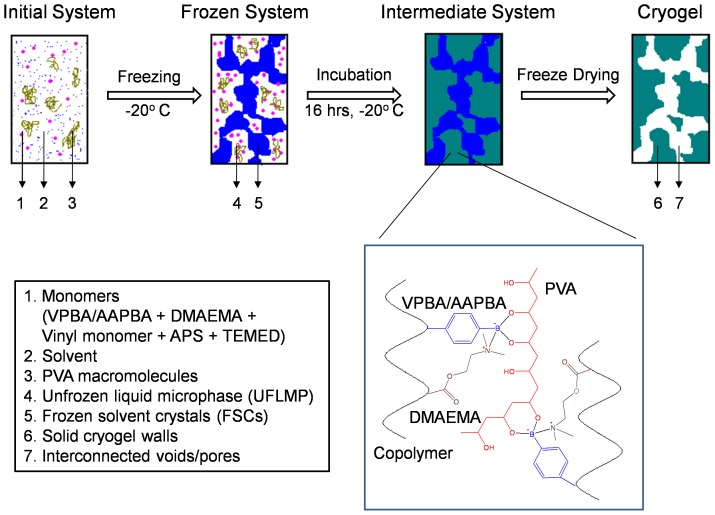
Conceptual depiction of phenylboronate containing copolymer (PBCC)-PVA cryogel synthesis. Freezing initiates the phase separation of the initial monomer-PVA solution into unfrozen liquid microphase (UFLMP) and frozen solvent crystals (FSCs). As a result UFLMP gets concentrated with monomers and PVA. Within UFLMP, APS and TEMED initiates the free radical polymerization of monomers into PBCC chains that get crosslinked *via* PVA upon attaining sufficient length. This results in solid cryogel walls separated by FSCs. Freeze drying sublimes FSCs leaving behind interconnected voids/pores.

**Figure 5:**
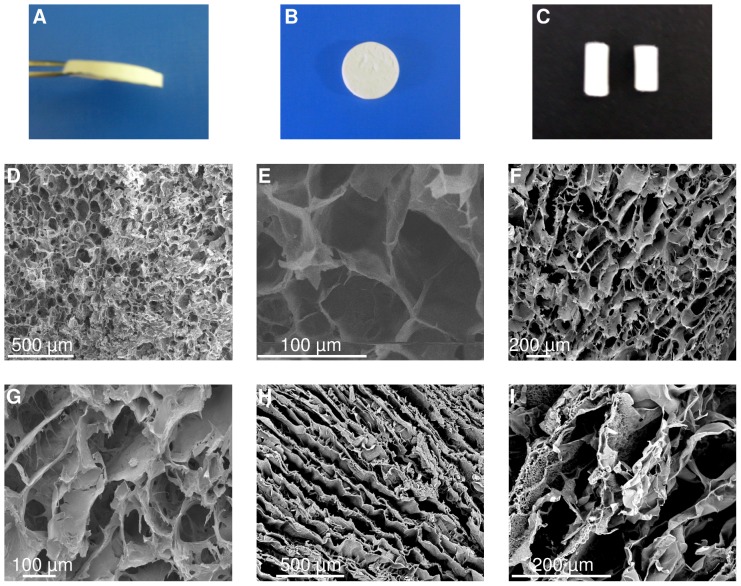
Phenylboronate containing copolymer (PBCC)-poly(vinylalcohol) (PVA) cryogels. Figs. 5A-C shows sheets and monoliths of NVP cryogels. SEM micrographs of NVP cryogels (Figs. 5D, E), AVP cryogels (Figs. 5F, G) and AADP cryogels (Figs. 5H, I) reveal their open macroporous structure.

Poly(NIPAAm-*co*-VPBA)-PVA (NVP cryogel), poly(AAm-*co*-VPBA)-PVA (AVP cryogel) and poly(AAm-*co*-AAPBA-*co*-DMAEMA)-PVA (AADP cryogel) cryogel sheets and monoliths are synthesized *via* free radical polymerization in presence of PVA at sub-zero temperatures. Following freezing, the initial uniform gel precursor system (monomers, ammonium persulfate (APS), *N,N,N',N*'-tetramethylethylenediamine (TEMED) and PVA) gets phase separated into unfrozen liquid microphase (UFLMP) and the frozen solvent crystals (FSCs). Due to cryoconcentration UFLMP gets concentrated with precursors. APS and TEMED initiate the copolymerization of monomers in to PBCC. On attaining sufficient length, the PBCC chains get crosslinked by PVA *via* intermolecular multipoint monodiols resulting in solid cryogel walls in the place of UFLMP. The frozen solvent crystals are continuous throughout the system which gets sublimed upon freeze drying and thereby resulting in an open porous system (Fig. 4). Scanning electron microscope (SEM) analysis revealed the open porous structure of the cryogels with thick and rough pore wall morphology. NVP cryogel (Fig. 5A-E) and AVP cryogel (Fig. 5F, G) showed typical open porous structure with approximate pore size varying from 40–150 µm and 50–200 µm, respectively. AADP cryogel (Fig. 5H, I) revealed an unconventional microstructure with thick and longitudinal pore wall. The pores remained macroporous and interconnected.

### Compression testing analysis of cryogels

In compression testing studies, AVP cryogel showed highest elastic modulus (*λ*) of about 7.195 MPa followed by AADP (*λ*  = 3.895 MPa) and NVP (*λ*  = 2.172 MPa) cryogels (Fig. S3). The superior mechanical properties of PBCC-PVA cryogels over conventional poly(NIPAAm) and poly(AAm) cryogels (with *λ* usually in the range of KPa [6]) is due to the presence of PVA which is known to improve mechanical strength of cryogels. The presence of acrylamide as the monomer that constitutes majority of PBCC accounts for the higher *λ* of AVP cryogel compared to NVP cryogel. The higher *λ* of AVP cryogel when compared to AADP cryogel may suggest that VPBA results in stronger interactions and therefore crosslinking with polyols as compared to AAPBA.

### Fructose induced disintegration of cryogels

**Figure 6:**
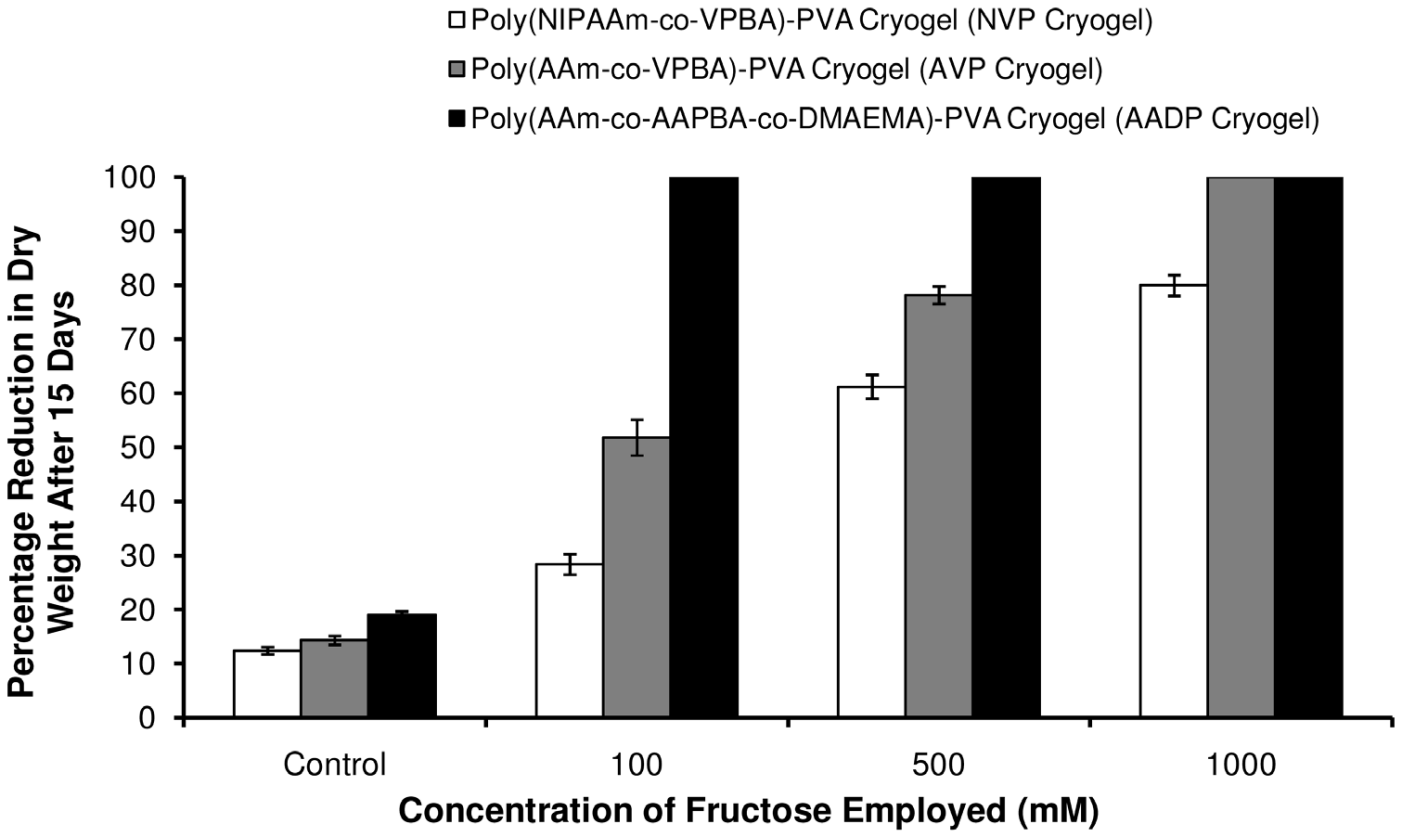
Fructose induced disintegration of phenylboronate containing copolymer (PBCC)-poly(vinylalcohol) (PVA) cryogels. Fig.6 plots the percentage reduction in dry weights of different PBCC-PVA cryogels after 15 days in response to three different fructose concentrations (100 mM, 500 mM and 1000 mM). The cryogels treated with fructose have exhibited significant amounts of degradation compared to controls i.e., cryogels treated with phosphate buffer devoid of monosaccharides. AADP cryogel has showed superior degradability even at low fructose concentrations (100 mM) followed by AVP and NVP cryogels, respectively.

The gel-sol disintegration of PBCC-PVA cryogels was evaluated by employing 100 mM, 500 mM and 1 M fructose-buffer solutions (Fig. 6). The percentage reduction in cryogel dry weights after 15 days of fructose treatment is presented in Table S1. All the cryogels showed significant amounts of fructose induced disintegration when compared to controls i.e., cryogels in buffer without any sugar. AADP cryogel, in particular, showed 100% disintegration within 15 days in response to fructose concentrations as low as 100 mM. The relatively slow disintegration of cryogels when compared to their hydrogel counterparts is most probably due to the extremely high concentrations of polymer at the cryogel walls as a result of cryoconcentration. The higher rates of disintegration exhibited by AADP cryogels compared to others, shows that the AAPBA-polyol interactions possess more reversibility characteristics as compared to VPBA-polyol interactions. Moreover the tertiary amine of DMAEMA may favor and stabilize the phenylboronate-fructose interactions at physiological pH. The relatively lower percentage reduction in dry weights exhibited by NVP cryogels when compared to AVP cryogels is probably due to the presence of NIPAAm in the copolymer chains of the former. Since the temperature used in disintegration studies is 37°C, it might have presented hydrophobic environment for the poly(NIPAAm-*co*-VPBA) chains in the cryogel slowing down their dissolution.

### Cell culture using 3-D cryogel scaffolds

**Figure 7:**
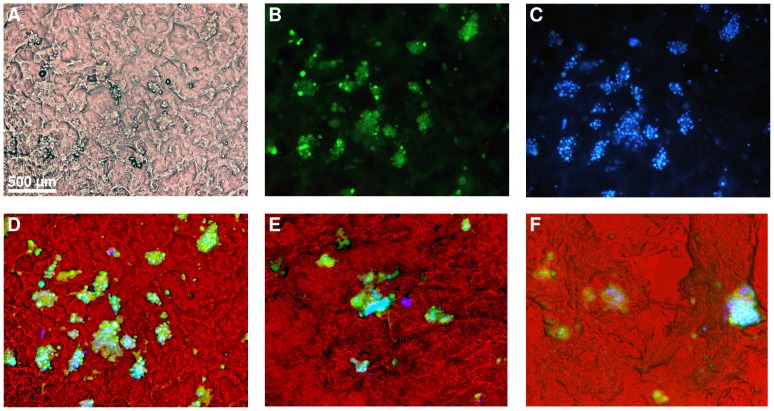
FDA/DAPI double staining of cryogel sections after 12 days in culture. Fig. 7A shows the bright field image of AVP cryogel with FDA/DAPI double stained NIH3T3 fibroblasts at day 12. Fig. 7B shows FDA stained viable cells (greenish fluorescence) and Fig. 7C shows DAPI stained viable and non-viable cells (bluish fluorescence) in AVP cryogel. Fig. 7D is a merged RGB image of Figs. 7A-C, respectively where cyan color indicates cell viability while non-viable cells show blue fluorescence alone. Figs. 7E, F are merged RGB images of double stained AADP and NVP cryogels, respectively (day 12). The merged images (Figs. 7D-F) of all the three cryogels are rich in cyan colored portions indicating their ability to support cell adhesion and viability.

**Figure 8:**
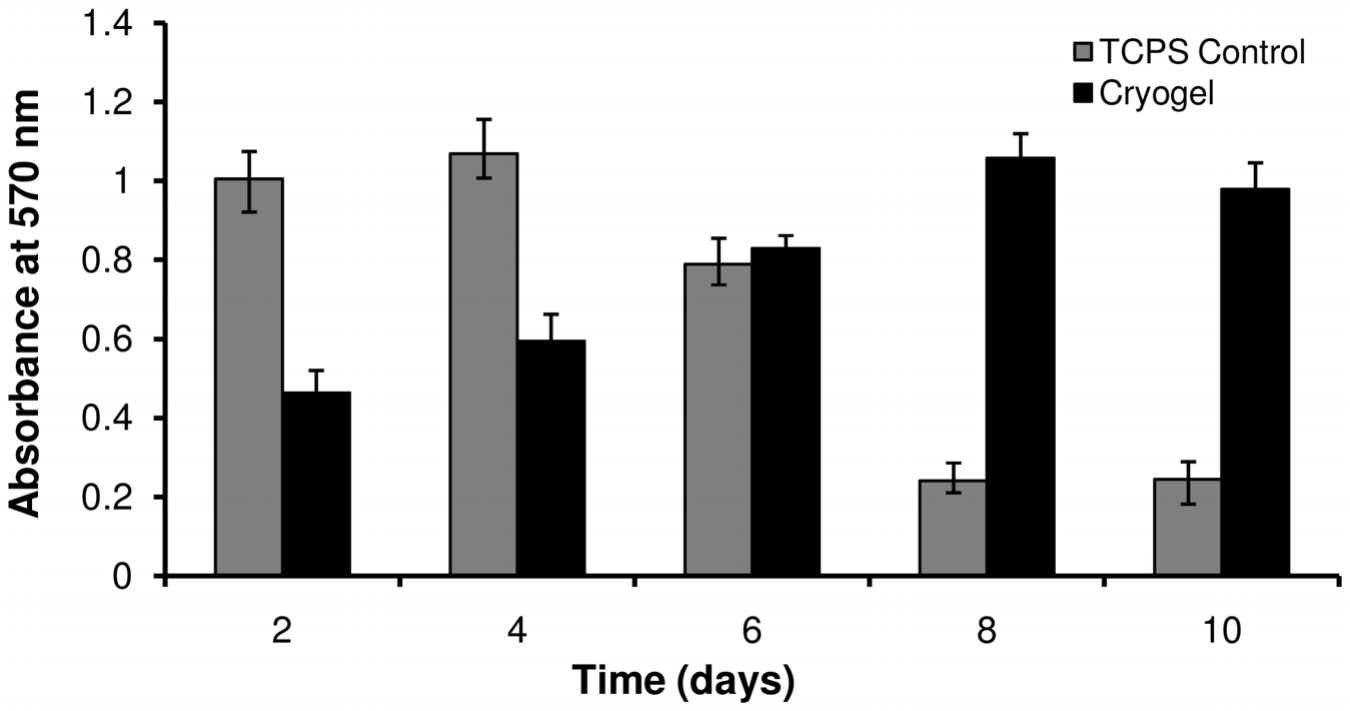
MTT assay of NIH3T3 fibroblasts cultured on AADP cryogels. Fibroblasts cultured on tissue culture polystyrene (TCPS) plates have served as controls. The fibroblasts in cryogel sections have recorded relatively low levels of metabolic activity during the first few days as compared to the controls. From day 6 onwards, cryogels have displayed good levels of cell metabolic activity whereas controls have recorded a significant decline in cell activity. This indicates the ability of cryogels to offer high surface area for the growing cells as compared to 2-D controls and further demonstrates their support for cell growth and viability.

NIH3T3 fibroblast cells (5.5×10^4^ cells/ml) suspended in DMEM were directly seeded on to cylindrical cryogel sections and incubated in humidified 5% CO_2_ environment. At day 12, the cryogel sections were double stained with fluorescein diacetate (FDA) and 4',6-diamidino-2-phenylindole (DAPI) to assess cell viability. FDA stains only viable cells which fluoresce, brilliant green under blue light whereas DAPI stains both viable and non-viable cells which fluoresce, blue under UV-light of a fluorescent microscope. Therefore in a merged RGB image of double stained cells, greenish cyan portions indicate viable cells whereas isolated blue portions indicate non-viable cells. The RGB image of AVP cryogel section with cells at day 12 merged from the individual bright field image of scaffold (Fig. 7A), FDA fluorescent image of viable cells (Fig. 7B) and DAPI fluorescent image of all cells (Fig. 7C), respectively was shown in Fig. 7D. The image contains cyan rich portions and negligible isolated blue portions indicating good cell viability even at day 12. Similarly the merged RGB images of AADP (Fig. 7E) and NVP cryogels (Fig. 7F) also showed good amounts of cell viability with negligible or no dead cells. In order to further assess the viability and proliferation of cells cultured on PBCC-PVA cryogels, 3-(4,5-dimethylthiazol-2-yl)-2,5-diphenyltetrazolium bromide (MTT) assay was carried out on fibroblast-seeded AADP cryogel sections on alternate days for 10 days. The results obtained for cryogels were compared with those of controls i.e., TCPS dishes seeded with same number of cells (Fig. 8). During the first few days (up to day 4) cell metabolic activity was low in case of cryogels when compared to 2-D controls. In the later days, the cryogels recorded good amounts of cell activity whereas controls showed a decline. This indicates the high surface area available for cells in the cryogel scaffolds when compared to 2-D controls.

## Discussion

Amine assisted PBCC-polyol interactions have been successfully utilized to develop phase transformable scaffold materials to deal with cells and tissues at physiological pH. The present study further confirms that the copolymerization of a tertiary amine and a phenylboronate ligand is the most convenient way to place the former proximal to boronate moiety in order to achieve boronate-di/polyol interactions at physiological pH. *N,N*-dimethylaminopropyl-acrylamide (DMAPAAm) is the most commonly used amine to achieve the above mentioned objective [20], [22], [23], [27]. We showed that DMAEMA can serve as a successful alternative to DMAPAAm in promoting boronate-di/polyol interactions at neutral pH. The amino group of DMAEMA acts as Lewis base and donates a pair of electrons to the vacant *p*-orbital of boron, which becomes tetrahedral, and prevents the surrounding water molecules from doing the same. As a result tetrahedral anionic form of boronate becomes dominant in the copolymer structure even at pH close to neutrality which ultimately results in reversible interactions with di/polyols. We have synthesized three phenylboronate-amine containing terpolymers which formed complex gels with PVA. The gels have exhibited monosaccharide sensitive gel-sol phase transition.

For the first time, the applicability of such PBCC-PVA hydrogels as cell culture substrates has been investigated. The peculiar aggregation of fibroblasts at specific positions over the hydrogel just after cell seeding probably suggests the existence of gel portions with pendant phenylboronates that were not engaged in reversible interactions with PVA diols. Such portions may result from inhomogeneous mixing of terpolymer and PVA solutions. Since anionic phenylboronate exhibit affinity for certain cell surface carbohydrate moieties [16], [17], the cells might get migrated to and aggregated at these portions. This particular property of PBCC-PVA hydrogels may present them as novel and interesting candidate materials for spheroid culture. The fibroblasts have showed good amounts of viability and proliferation once they get started to spread out on the gels. On achieving confluence, the cells have been harvested from the hydrogels by exploiting their fructose inducible gel-sol phase reversibility. The release of cell monolayer at the edges of the gel in the initial stages of fructose addition shows the potential of these hydrogels for cell sheet engineering applications. The chances of obtaining intact cell sheet may largely depend on the type of monomer that constitutes the majority of terpolymer backbone, the concentration and ionic strength of the monosaccharide solution administered as well as the time period of administration. Prolonged fructose treatment, for about 35–40 minutes, resulted in recovery of individual cells and small clumps. Owing to their biocompatibility, mild synthesis procedure and on demand gel to sol phase transition characteristics, the hydrogels may also have potential cell immobilization applications.

The PBCC-PVA cryogels have exhibited an open macroporous structure, good mechanical properties and a considerable degree of fructose induced disintegration. *In vitro* cell culture studies have revealed that the cryogels support cell adhesion, viability and proliferation. This represents a firm step towards incorporating bioerosion like properties into synthetic non-degradable 3-D tissue engineering scaffolds. Such scaffolds allow better control over *in vitro* cell cultivation in three dimensions as compared to conventional biodegradable scaffolds since one can regulate the scaffold disintegration and therefore tissue in-growth by varying the type and concentration of monosaccharide being administered as well as its time of administration. Besides cell sheet and tissue engineering applications, the materials and scaffolds described in our study may also serve as glucose sensitive drug delivery and saccharide recognition systems.

## Supporting Information

Figure S1 **Lower Critical Solution Temperature (LCST) of poly(N-isopropylacrylamide) (pNIPAAm) and poly(N-isopropylacrylamide-co-vinylphenylboronate-co-N,N-dimethylaminoethylmethacrylate) (NVDT).** The plot presents LCST curves of 0.5% w/v solutions of pNIPAAm (-▪-) and NVDT (-•-). The LCST was measured by taking the absorbance at 580 nm of 0.5% w/v polymer solutions at different temperatures.
(PDF)

Figure S2 **FT-IR spectra of poly(** ***N*** **-isopropylacrylamide-** ***co*** **-vinylphenylboronate-** ***co*** **-** ***N,N*** **-dimethylaminoethylmethacrylate) (NVDT) and the constituent monomers.** FT-IR characterization of NVDT has confirmed the successful copolymerization of the respective monomers. Absorbance of amide-carbonyl group at 1638.28 cm^−1^ and N-H bending at 1550.47 cm^−1^ are the characteristic peaks of *N*-isopropylacrylamide (NIPAAm). Appearance of a small peak at 1715.18 cm^−1^ for C = O stretching confirm the copolymerization of *N,N*-dimethylaminoethylmethacrylate (DMAEMA). Further, two characteristic absorption bands for –N(CH_3_)_2_ of DMAEMA are observed at 2971.45 cm^−1^ and 2925.30 cm^−1^. A broad band of absorbance has appeared at 3423.22 cm^−1^ for the hydrogen bonded OH-groups of 4-vinylphenylboronate (VPBA). A benzene ring vibration at 1463.39 cm^−1^, B-O stretching at 1367.86 cm^−1^ and B-OH at 1130.66 cm^−1^ confirms VPBA as a part of the terpolymer.
(PDF)

Figure S3 **Stress-strain behavior of phenylboronate containing copolymer (PBCC)-poly(vinylalcohol) (PVA) cryogels.** Figs. S3a-c shows the stress-strain behavior of NVP, AVP and AADP cryogels, respectively, under uniaxial compression with 100 kN load cell under displacement control at the rate of 1 mm/min. The modulus of elasticity (*λ*) for each cryogel sample is calculated from the elastic deformation portion of the respective stress-strain graph. PBCC-PVA cryogels have showed superior mechanical strength compared to conventional poly(*N*-isopropylacrylamide) and poly(acrylamide) cryogels due to the presence of PVA. The superior mechanical properties of AVP (*λ*  =  7.195 MPa) and AADP (*λ*  =  3.895 MPa) cryogels compared to that of NVP (*λ*  = 2.172 MPa) cryogels can be attributed to the presence of acrylamide as copolymer backbone. The elastic moduli of AVP and AADP cryogels suggest that 4-vinylphenylboronic acid-polyol interactions are relatively stronger than *N*-acryloyl-3-aminophenylboronic acid-polyol interactions.
(PDF)

Table S1 Fructose induced disintegration of synthesized PCC-PVA cryogels after 15 days. The cryogels treated with phosphate buffer have served as controls. Fructose treated cryogels have exhibited significant amounts of disintegration as compared to controls. AADP cryogels have recorded highest levels of fructose-responsiveness followed by AVP and NVP cryogels, respectively.
(PDF)
