# Supplementary material for: Monosaccharide-Responsive Phenylboronate-Polyol Cell Scaffolds for Cell Sheet and Tissue Engineering Applications
Source: PLoS One. 2013 Oct 22;8(10):e77861. doi: 10.1371/journal.pone.0077861 (PMC3805603; doi:10.1371/journal.pone.0077861)
Supplement: Figure S1 — Lower Critical Solution Temperature (LCST) of poly(N-isopropylacrylamide) (pNIPAAm) and poly(N-isopropylacrylamide-co-vinylphenylboronate-co-N,N-dimethylaminoethylmethacrylate) (NVDT). The plot presents LCST curves of 0.5% w/v solutions of pNIPAAm (-▪-) and NVDT (-•-). The LCST was measured by taking the absorbance at 580 nm of 0.5% w/v polymer solutions at different temperatures. (PDF) [file pone.0077861.s001.pdf]

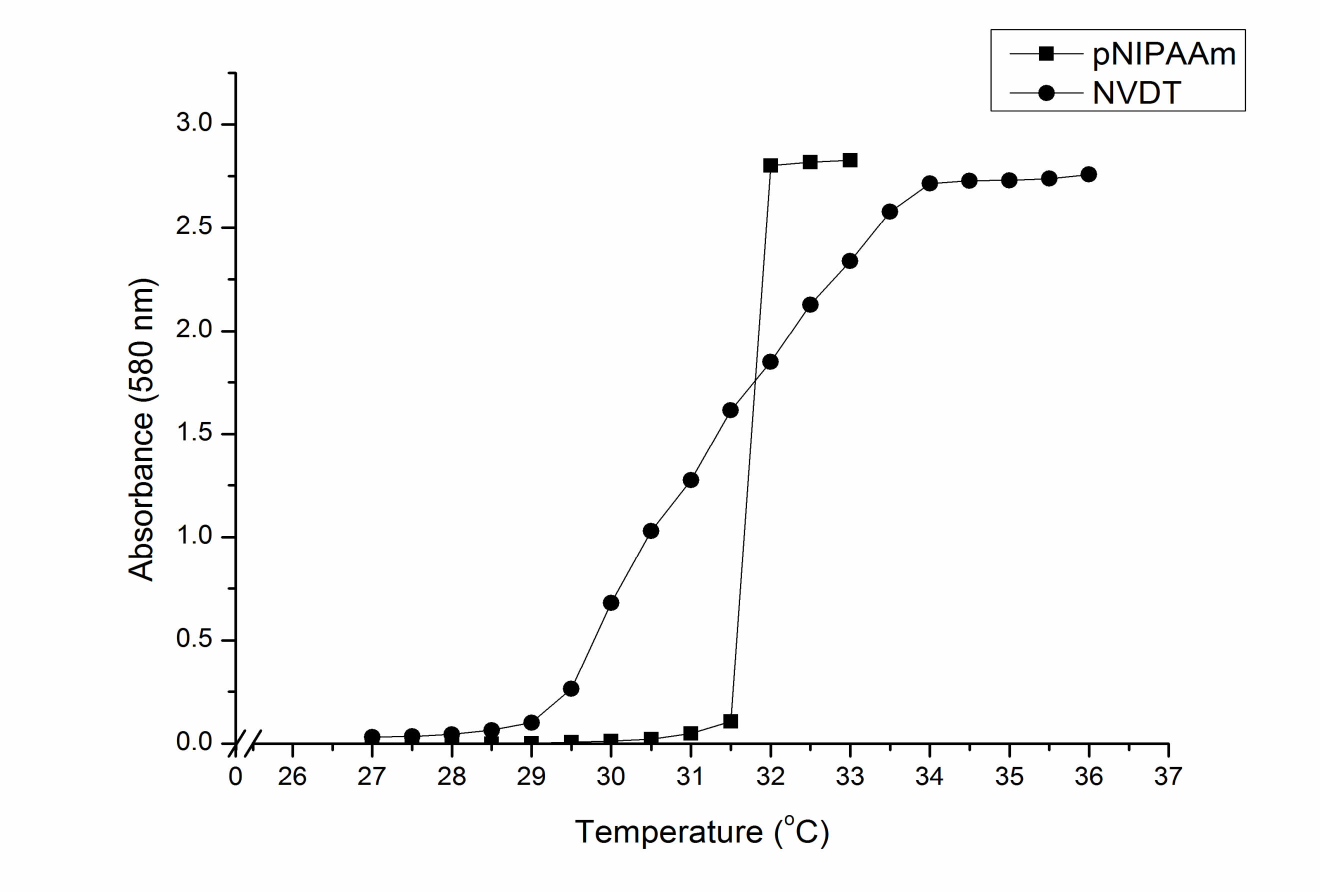

Figure S1: Lower Critical Solution Temperature (LCST) of poly(N-isopropylacrylamide) (pNIPAAm) and poly(N-isopropylacrylamide-co-vinylphenylboronate-co-N,N-dimethylaminoethylmethacrylate) (NVDT). The plot presents LCST curves of 0.5% w/v solutions of pNIPAAm (-■-) and NVDT (-●-). The LCST was measured by taking the absorbance at 580 nm of 0.5% w/v polymer solutions at different temperatures.
