# Supplementary material for: Monosaccharide-Responsive Phenylboronate-Polyol Cell Scaffolds for Cell Sheet and Tissue Engineering Applications
Source: PLoS One. 2013 Oct 22;8(10):e77861. doi: 10.1371/journal.pone.0077861 (PMC3805603; doi:10.1371/journal.pone.0077861)
Supplement: Figure S2 — FT-IR spectra of poly( N -isopropylacrylamide- co -vinylphenylboronate- co - N,N -dimethylaminoethylmethacrylate) (NVDT) and the constituent monomers. FT-IR characterization of NVDT has confirmed the successful copolymerization of the respective monomers. Absorbance of amide-carbonyl group at 1638.28 cm−1 and N-H bending at 1550.47 cm−1 are the characteristic peaks of N-isopropylacrylamide (NIPAAm). Appearance of a small peak at 1715.18 cm−1 for C = O stretching confirm the copolymerization of N,N-dimethylaminoethylmethacrylate (DMAEMA). Further, two characteristic absorption bands for –N(CH3)2 of DMAEMA are observed at 2971.45 cm−1 and 2925.30 cm−1. A broad band of absorbance has appeared at 3423.22 cm−1 for the hydrogen bonded OH-groups of 4-vinylphenylboronate (VPBA). A benzene ring vibration at 1463.39 cm−1, B-O stretching at 1367.86 cm−1 and B-OH at 1130.66 cm−1 confirms VPBA as a part of the terpolymer. (PDF) [file pone.0077861.s002.pdf]

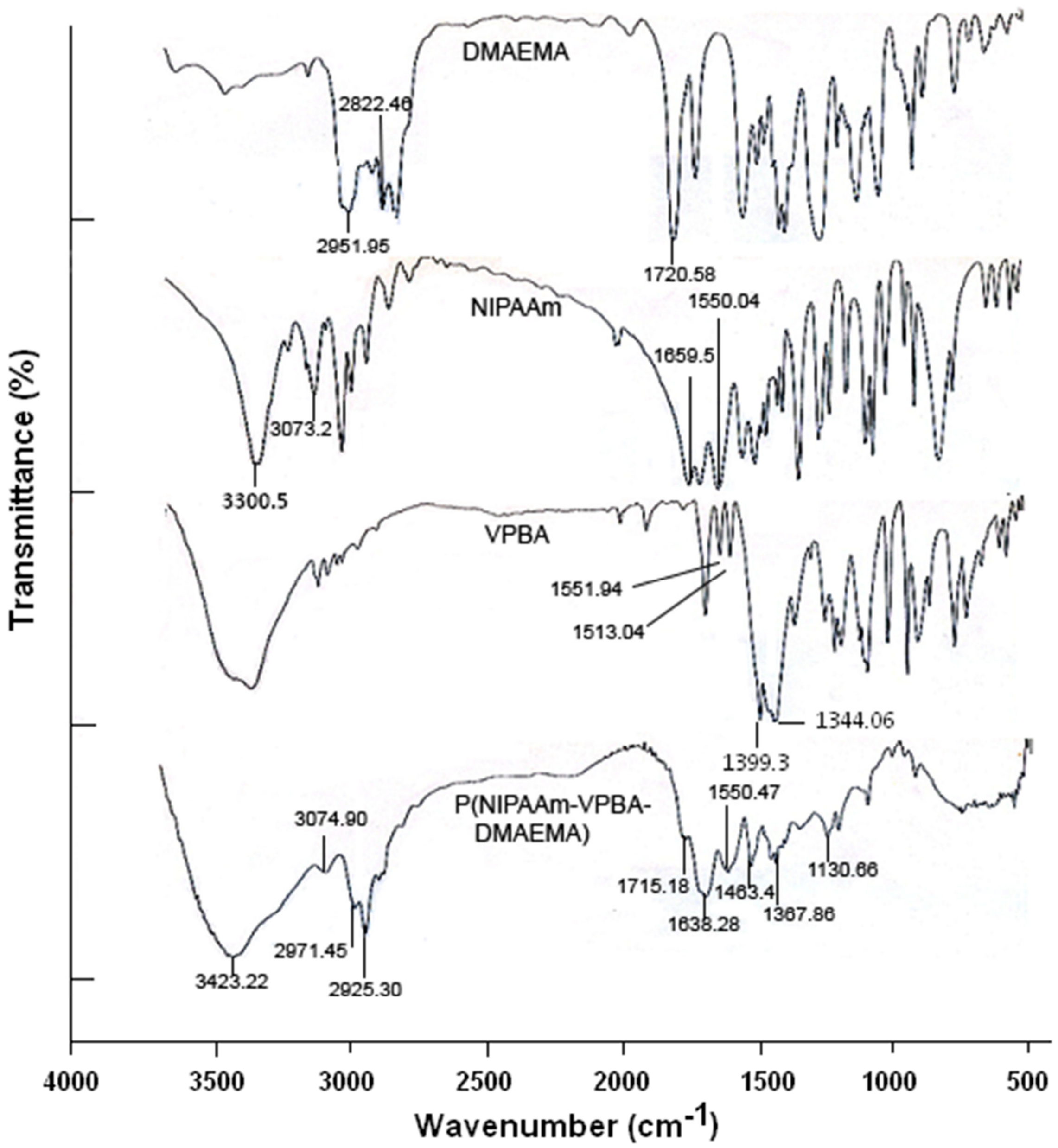

Figure S2. FT-IR spectra of poly(*N*-isopropylacrylamide-*co*-vinylphenylboronate-*co*-*N,N*-dimethylaminoethylmethacrylate) (NVDT) and the constituent monomers. FT-IR characterization of NVDT has confirmed the successful copolymerization of the respective monomers. Absorbance of amide-carbonyl group at 1638.28 cm^{-1} and N-H bending at 1550.47 cm^{-1} are the characteristic peaks of *N*-isopropylacrylamide (NIPAAm). Appearance of a small peak at 1715.18 cm^{-1} for C=O stretching confirm the copolymerization of *N,N*-dimethylaminoethylmethacrylate (DMAEMA). Further, two characteristic absorption bands for $–N(CH_3)_2$ of DMAEMA are observed at 2971.45 cm^{-1} and 2925.30 cm^{-1}. A broad band of absorbance has appeared at 3423.22 cm^{-1} for the hydrogen bonded OH-groups of 4-vinylphenylboronate (VPBA). A benzene ring vibration at 1463.39 cm^{-1}, B-O stretching at 1367.86 cm^{-1} and B-OH at 1130.66 cm^{-1} confirms VPBA as a part of the terpolymer.
