# Supplementary material for: Monosaccharide-Responsive Phenylboronate-Polyol Cell Scaffolds for Cell Sheet and Tissue Engineering Applications
Source: PLoS One. 2013 Oct 22;8(10):e77861. doi: 10.1371/journal.pone.0077861 (PMC3805603; doi:10.1371/journal.pone.0077861)
Supplement: Figure S3 — Stress-strain behavior of phenylboronate containing copolymer (PBCC)-poly(vinylalcohol) (PVA) cryogels. Figs. S3a-c shows the stress-strain behavior of NVP, AVP and AADP cryogels, respectively, under uniaxial compression with 100 kN load cell under displacement control at the rate of 1 mm/min. The modulus of elasticity (λ) for each cryogel sample is calculated from the elastic deformation portion of the respective stress-strain graph. PBCC-PVA cryogels have showed superior mechanical strength compared to conventional poly(N-isopropylacrylamide) and poly(acrylamide) cryogels due to the presence of PVA. The superior mechanical properties of AVP (λ = 7.195 MPa) and AADP (λ = 3.895 MPa) cryogels compared to that of NVP (λ = 2.172 MPa) cryogels can be attributed to the presence of acrylamide as copolymer backbone. The elastic moduli of AVP and AADP cryogels suggest that 4-vinylphenylboronic acid-polyol interactions are relatively stronger than N-acryloyl-3-aminophenylboronic acid-polyol interactions. (PDF) [file pone.0077861.s003.pdf]

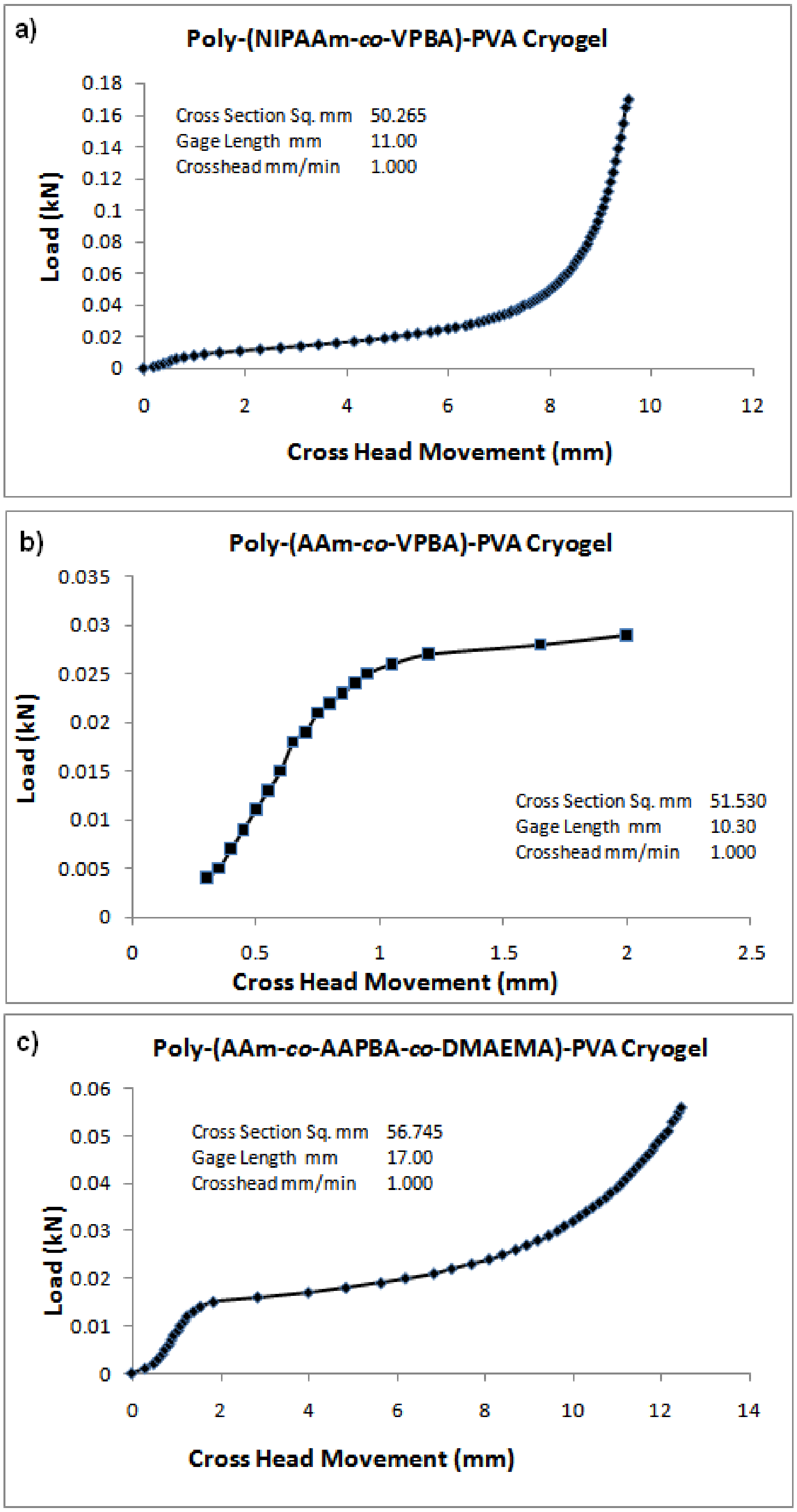

Figure S3. Stress-strain behavior of Phenylboronate containing copolymer (PBCC)-poly(vinylalcohol) (PVA) cryogels. Figs. S3a-c show the stress-strain behavior of NVP, AVP and AADP cryogels, respectively, under uniaxial compression with 100 kN load cell under displacement control at the rate of 1 mm/min. The modulus of elasticity (λ) for each cryogel sample is calculated from the elastic deformation portion of the respective stress-strain graph. PBCC-PVA cryogels have showed superior mechanical strength compared to conventional poly(*N*-isopropylacrylamide) and poly(acrylamide) cryogels due to the presence of PVA. The superior mechanical properties of AVP (λ = 7.195 MPa) and AADP (λ = 3.895 MPa) cryogels compared to that of NVP (λ =2.172 MPa) cryogels can be attributed to the presence of acrylamide as copolymer backbone. The elastic moduli of AVP and AADP cryogels suggest that 4-vinylphenylboronic acid-polyol interactions are relatively stronger than *N*-acryloyl-3-aminophenylboronic acid-polyol interactions.
