# Supplementary material for: Monosaccharide-Responsive Phenylboronate-Polyol Cell Scaffolds for Cell Sheet and Tissue Engineering Applications
Source: PLoS One. 2013 Oct 22;8(10):e77861. doi: 10.1371/journal.pone.0077861 (PMC3805603; doi:10.1371/journal.pone.0077861)
Supplement: Table S1 — Fructose induced disintegration of synthesized PCC-PVA cryogels after 15 days. The cryogels treated with phosphate buffer have served as controls. Fructose treated cryogels have exhibited significant amounts of disintegration as compared to controls. AADP cryogels have recorded highest levels of fructose-responsiveness followed by AVP and NVP cryogels, respectively. (PDF) [file pone.0077861.s004.pdf]

Table 1. Fructose induced disintegration of synthesized PCC-PVA cryogels after 15 days.

	Percentage reduction in cryogel dry weight after 15 days in response to			
**Cryogel**	**Buffer**	**Fructose (100 mM)**	**Fructose (500 mM)**	**Fructose (1 M)**
**NVP**[A]	12.45±0.67	28.41±2.20	61.23±2.0	79.98±1.93
**AVP**[B]	14.37±0.81	51.84±3.34	78.17±1.64	100
**AADP**[C]	19.07±0.68	100	100	100

The cryogels treated with phosphate buffer have served as controls. Fructose treated cryogels have exhibited significant amounts of disintegration as compared to controls. AADP cryogels have recorded highest levels of fructose-responsiveness followed by AVP and NVP cryogels respectively.

[A]NVP : Poly(NIPAAm-co-VPBA)-PVA Cryogel
[B]AVP : Poly(AAm-co-VPBA)-PVA Cryogel
[C]AADP : poly(AAm-co-AAPBA-co-DMAEMA)-PVA
